# Development of a New Questionnaire to Assess Parental Perceived Barriers When Promoting Healthy Eating Habits in Young Children: First Findings

**DOI:** 10.3390/nu15214672

**Published:** 2023-11-04

**Authors:** Ana Isabel Gomes, Ana Isabel Pereira, Patrícia Canhoto Nogueira, Luísa Barros

**Affiliations:** 1 Faculty of Psychology, University of Lisbon, Alameda da Universidade, 1649-013 Lisboa, Portugal; patriciacnogueira@campus.ul.pt; 2 CICPSI, Faculty of Psychology, University of Lisbon, Alameda da Universidade, 1649-013 Lisboa, Portugal; aipereira@psicologia.ulisboa.pt (A.I.P.); lbarros@psicologia.ulisboa.pt (L.B.)

**Keywords:** barriers, parents, preschool children, child’s eating habits, scale development, psychometric study

## Abstract

Social cognitive models suggest a crucial role played by perceived barriers in promoting healthy behaviors, including healthy eating. We aimed to develop a new questionnaire to assess parental perceived barriers to healthy feeding in young children and perform the instrument’s preliminary psychometric evaluation. The initial pool of items was developed based on reviews and qualitative studies. First, we conducted an online, descriptive, cross-sectional study with 278 parents of 2–6-year-old children to examine its factorial structure and internal consistency. Then, a second study with 168 parents from a similar population assessed convergent/discriminant and known-groups validity. The exploratory factorial analysis confirmed the scale’s theoretical structure. Five scales were found: Child-Related Barriers, Parent-Related Barriers—Vegetables and Fruit, Parent-Related Barriers—Added Sugars, Social Context-Related Barriers, and Cost-Related Barriers. All scales presented adequate reliability. We found weak to moderate, negative, and significant correlations between child- and parent-related barriers regarding vegetables and fruits, feeding practices to promote children’s eating self-regulation, and food parenting self-efficacy. Additionally, parents who perceived their children as easy and well-regulated reported significantly fewer child-related barriers than parents with poorly self-regulated and inhibited children. The results support the instrument’s preliminary psychometric adequacy regarding its validity and reliability and corroborate earlier empirical studies about the main parental barriers when promoting young children’s healthy eating habits.

## 1. Introduction

Parents play a critical role in young children’s healthy eating behaviors and routines by shaping the early eating environment and using various feeding practices [1]. Most parents have reasonable knowledge about what is a healthy diet; they want their children to eat healthy and nutritious foods [2], regularly eat vegetables [3], or restrict their added sugar intake [4]. However, parents’ intention to promote healthy eating or change unhealthy food choices and behaviors is often hindered by several objective or subjective barriers to performing the relevant practices or behaviors [5].

Bandura’s social cognitive theory [6] defines barriers to health behavior as socio-structural impediments that can be individual and part of one’s self-efficacy assessment or external, such as the organization of health services. Considering the health belief model [7], perceived barriers involve the subjective perception of the obstacles or difficulties in performing specific behaviors or the anticipated negative consequences of these behaviors [8]. These barriers may discourage parents from getting involved in promoting healthy eating or be an obstacle to maintaining healthy routines and practices. Identifying these barriers is crucial to understanding how they interfere with parents’ intentions and affect their practices and child outcomes. Several food parenting interventions include strategies to support parents in identifying and overcoming these barriers [9,10].

Several studies have consistently identified barriers faced by parents and related to their intentions and behaviors to promote healthy eating in preschool children. A systematic review by Pocock et al. [11] about parents’ healthy behaviors for preventing overweight and obesity organizes these barriers into three main categories, according to the socioecological model [12]: individual, relational, and contextual factors. Contextual and external factors were often identified, such as the cost of healthy foods, lack of time or availability of healthy foods [5,11,13,14,15,16], or the influence of media and publicity [13,14,15]. Also often considered are parental factors (cooking skills, nutritional knowledge) [5,17,18] or child-related factors (e.g., food preferences, soliciting sugary foods, or eating behavior) [11,14,15,19]. Hingle and colleagues [3] studied the barriers to vegetable parenting practices and identified three main factors: the child dislikes vegetables, the parent dislikes vegetables, and the cost of vegetables, including the economic value and the work to prepare them.

Most studies about barriers in the context of food parenting use a qualitative approach through individual interviews, focus groups, or open-ended questions [20]. Meanwhile, some questionnaires that target parental barriers were developed for specific feeding contexts. Baranowski et al.’s [21] questionnaire evaluates parents’ perceived barriers to promoting children’s vegetable intake with three dimensions, but these are restricted to vegetable parenting. Storfer-Isser and Musher-Eizenman [22] developed a brief measure of scarcity and fatigue as parents’ barriers to planning and preparing meals for their children. Darling et al. [23] proposed the Barriers to Child Weight Management scale to assess barriers parents face when trying to obtain and maintain a healthy weight status for children, containing four factors: parental disengagement, cost and the built environment, lack of family support, and family time constraints.

This study aimed to contribute to this domain by proposing a new instrument assessing parents’ perceived barriers to promoting their preschool child’s healthy eating. Similarly to other countries, the main issues in Portuguese young children’s diets are related to the low consumption of vegetables and fruit and high intake of added sugar in foods and beverages [24]. Thus, the instrument was developed considering the barriers and difficulties related to children’s intake of these three food groups. We propose to assess the instrument’s psychometric features regarding its validity and reliability; the convergent, discriminant, and known-groups validity of the measure will also be studied through correlations with parental self-efficacy to promote their child’s healthy eating habits, parental practices to promote children’s eating self-regulation, and the child’s temperament. Based on social cognitive theory, we anticipated that all types of barriers might be negatively related to parenting self-efficacy regarding the promotion of healthy eating among children. However, there may be differences in their magnitude due to perceived parental control (expectedly, stronger relationships with individual barriers than contextual ones). Earlier studies on the predictors of effective vegetable parenting practices support a possible significant negative correlation between parental use of practices to promote children’s self-regulation of food intake and child- and parent-related barriers [25]. We also hypothesized that parents who perceive their children as easy and well-regulated would report fewer child-related barriers than parents with poorly self-regulated or inhibited children [26,27].

## 2. Methods

### 2.1. Development of the Scale

The development of the Parental Perception of Children’s Healthy Feeding Barriers Questionnaire started with a literature review of earlier qualitative and quantitative studies about parental perceived barriers and challenges regarding the promotion and/or improvement of healthy eating habits in their children. Based on the work of Hingle et al. [3] and Baranowski et al. [21], as well as qualitative studies related to the topic of this study [5,14,15,17,28], the first pool of items related to parents’ barriers to the promotion of healthy eating in young children was created. Since Baranowski and colleagues’ [21] work was limited to the study of vegetable parenting practices, new items related to the promotion of fruit consumption and reduced consumption of foods with added sugars were also developed.

This pool of items was sent to two specialists in the field (a nutritionist and a psychologist experienced in research and intervention in this domain) to assess the relevance and suitability of the items. Subsequently, the most appropriate ones were selected for the final questionnaire. A semantic validation of these items was carried out through interviews with six parents, asking them to assess the relevance, difficulty of understanding, and adequacy of each item and to paraphrase the items in their own words to ascertain the level of comprehension. The wording of some items was improved to prepare the final list.

### 2.2. General Procedures

This study was approved by the Ethics and Deontology Committee of the Faculty of Psychology, University of Lisbon (approval ID: CD/4/1.5/2018). The scale’s psychometric evaluation was accomplished in two phases: a first study was carried out to confirm the theoretical structure of the scale through an exploratory factorial analysis (EFA) and assessment of its internal consistency, and a second study was performed to test convergent/discriminant and known-groups validity of the instrument, considering the structure confirmed in the first study. The evaluation protocols were administered to 406 parents of 2–6-year-old children, and the data collection occurred between 8 January and 29 April 2019 (Study 1) and between 10 May 2021 and 15 January 2022 (Study 2). Both studies were disseminated through social networks, and the evaluation protocol was available online through a Qualtrics platform link. The invitation to participate in the study included a consent form (informing participants about the study objectives and collaboration conditions) that parents read and agreed to before accessing the protocol.

## 3. Study 1: Descriptive Analysis of the Items, Factorial Structure of the Scale, and Reliability Analysis

### 3.1. Sample Characteristics

The evaluation protocol was completed by 278 parents of 2–6-year-old children (M = 3.80, SD = 1.30). Most participants were mothers (87.1%) with a higher education degree (80.6%), aged between 20 and 50 years old (M = 36.89; SD = 5.42).

### 3.2. Measures

Sociodemographic questionnaire: We collected data about the parents’ age, sex, level of education, kinship with the child, and the child’s age.

Parental Perception of Children’s Healthy Feeding Barriers Questionnaire: The 37-item version of the scale was answered on a five-point Likert scale (totally false to totally true) with higher values indicating a higher agreement about the existence/presence of a specific barrier.

### 3.3. Statistical Analysis

An exploratory factorial analysis of the instrument was conducted according to the following statistical procedures and criteria: (a) assessment of the sampling adequacy through Bartlett’s sphericity tests (*p*-value inferior to 0.05) and the Kaiser–Meyer–Olkin measure (above 0.70); (b) examination of the instrument’s factor structure through the principal components method and the Varimax rotation; (c) combination of the scree plot, the eigenvalue and Horn’s parallel analysis [29] to confirm the number of factors in the instrument’s structure, and; (d) verification of item loadings in each factor, with retention of all the items with factor loadings higher than 0.30 and differences between loadings in two or more factors superior to 0.10. The reliability of the scale’s new structure was then confirmed, with McDonald’s coefficient omega (ϖ) [30] (or Cronbach’s alpha coefficient (α) when the subscales had less than three items) and the inter-item correlation means (IICM). Internal consistency was considered good if alpha values exceeded 0.70 and IICM values were higher than 0.20.

### 3.4. Results

#### 3.4.1. Exploratory Factorial Analysis (EFA)

In the EFA, the scree plot and information regarding eigenvalues suggested the retention of five components. Nine items did not comply with the criteria for factor loadings, so we excluded them in a second analysis. The sample adequacy was verified by Bartlett’s tests of sphericity (χ2 = 3327.31, *p* < 0.000) and the Kaiser–Meyer–Olkin (KMO = 0.77) measure. The theoretical structure of the scale with five factors (Child-Related Barriers, nine items; Parent-Related Barriers—Vegetables and Fruit, six items; Parent-Related Barriers—Added Sugars, five items; Social Context-Related Barriers, six items; and Cost-Related Barriers, two items) was validated by the EFA and explained 52.9% of the total variance (Table 1).

#### 3.4.2. Internal Consistency of the Instrument and Correlation between Subscales

The subscales presented good internal consistency coefficients (between ϖ = 0.72 for Parent-Related Barriers—Added Sugars, and α = 0.95 for Cost-Related Barriers), except for the Social Context-Related Barriers scale (ϖ = 0.64). The IICM values were also adequate (between 0.25 for Social Context-Related Varriers subscale and 0.90 for the Cost-Related Barriers subscale). We also found mainly positive, significant, and weak to moderate correlations between the subscales (Pearson or Spearman correlation coefficients between 0.45 and 0.12, *p* < 0.05), except for the Social Context-Related Barriers scale associations with Child-Related Barriers and Parent-Related Barriers—Vegetables and Fruit scales, which were not related.

## 4. Study 2: Convergent/Discriminant and Known-Groups Validity of the Instrument

### 4.1. Sample Characteristics

In this study, 168 parents of young children participated, with a mean age of 36.90 years old (SD = 5.39), primarily mothers (92.9%) with a higher education degree (62.5%). Children were 2 to 6 years old (M = 4.51, SD = 1.16), and 54.2% were male. A small percentage of children had a chronic health condition (6.5%) or had received professional consultation for weight or nutritional issues (2.4%). 

### 4.2. Measures

Sociodemographic questionnaire: We collected general information about the children (age and sex, if the child had a chronic health condition or had received professional counseling due to weight or nutritional problems) and their parents (age and sex, education level, and kinship with the child).

Food parenting self-efficacy: The Parental Self-Efficacy for Children’s Healthy Diet Scale [31,32] was adopted to assess this dimension; this four-item scale aims to assess the extent to which parents are sure about their ability to promote the child’s intake of healthy foods and control the child’s intake of unhealthy foods. The items are answered on a five-point Likert scale (from not at all sure to absolutely sure), with higher values corresponding to higher parental self-efficacy. The good internal consistency of the instrument in its original version (α = 0.74; IICM = 0.35) [31,32] was confirmed with this sample (0.82; IICM = 0.53).

Parental feeding practices to promote children’s eating self-regulation: The Children’s Intake Self-Regulation Feeding Practices Scale [33] is an eight-item instrument that evaluates two kinds of parental practices that support children’s development of food intake self-regulation (e.g., teaching about the consequences of healthy and unhealthy food intake, and encouraging and helping the child to identify hunger and satiety cues). The items are answered on a five-point Likert scale (totally false to totally true), with higher values indicating more frequent use of feeding practices to promote children’s eating self-regulation. Both scales showed good internal consistency (teaching about eating consequences: ϖ = 0.72; IICM = 0.42; prompting for eating self-regulation: ϖ = 0.71; IICM = 0.37) in their study validation [33].

Child’s temperament: We asked parents to read three brief descriptions of three temperament types (poorly self-regulated or under-controlled, inhibited or reactive, and easy or well-adjusted) and choose which better describes their child. This temperament assessment was based on previous studies [34,35,36].

### 4.3. Statistical Analysis

The instrument’s convergent/discriminant validity was evaluated through correlational analysis between the Parental Perception on Children’s Healthy Feeding Barriers Questionnaire identified scales, Parental Self-efficacy for Children’s Healthy Diet Scale, and Children’s Intake Self-Regulation Feeding Practices Scale. We also studied differences regarding child-related barrier scores in parents of children with different temperament types (i.e., known-groups validity); the three groups (i.e., easy or well-regulated, inhibited or poorly self-regulated or under-controlled) were previously compared regarding children’s sex and age. Statistical significance of the tests (Pearson, Spearman, one-way ANOVA) was achieved for *p* < 0.05; the Bonferroni adjustment was applied for pairwise comparisons.

### 4.4. Results 

#### 4.4.1. Convergent and Discriminant Analysis

Correlation analysis (Table 2) showed that parents with higher food parenting self-efficacy reported fewer child-related and parent-related barriers for vegetable and fruit intake. We also found weak, significant, and negative correlations between both parental feeding practices to promote children’s eating self-regulation subscales and the child-related and parent-related barriers—vegetables and fruits subscales, and between teaching about eating consequences practices and parent-related barriers—added sugars subscale.

The three temperament groups did not significantly differ regarding children’s sex (χ^2^_(2)_ = 2.049; *p* = 0.359) and age (F_(2,165)_ = 1.320, *p* = 0.270). Parental reporting of child-related barriers significantly differed according to their child’s temperament type (F_(2,165)_ = 4.509, *p* = 0.012). Multiple comparisons revealed that parents who perceived their child as easy or well-regulated (2.45 ± 0.82) reported significantly fewer child-related barriers than those who perceived their children as poorly self-regulated or under-controlled (2.85 ± 0.82; *p* = 0.024) and inhibited (2.94 ± 0.81, *p* = 0.034). There was no significant differences regarding the report of child-related barriers between parents who perceived their child as poorly self-regulated and inhibited (*p* = 0.849). 

#### 4.4.2. Descriptive Analysis of the Sample

We calculated the subscale scores through the item answers’ mean (a possible score range between 1 and 5 on each scale). Overall, parents reported moderate levels of all barriers (child-related barriers: m = 2.76, sd = 0.82; parent-related barriers—added sugars: m = 2.49, sd = 0.66; social context-related barriers: m = 2.83, sd = 0.57; cost-related barriers: m = 2.30, sd = 0.96), except for parent-related barriers—vegetables and fruit (M = 1.83, SD = 0.60).

## 5. Discussion

The current work aimed to develop a new instrument to assess barriers faced by parents related to their efforts to promote healthy eating in young children and study its validity and reliability. Identifying specific barriers regarding their child’s feeding can help researchers and clinicians optimize feeding strategies proposed to parents during interventions. The first findings regarding the psychometric features of the instrument confirmed its adequacy regarding validity and reliability. The factorial analysis corroborated the main types of barriers identified by the literature [5,11,14,15]: child-related barriers, parent-related barriers, context-related barriers, and cost-related barriers. Also, the scales presented good internal reliability and weak to moderate correlations between them, confirming the relative independence of the dimensions.

In our study, the EFA suggested two dimensions for parent-related barriers: low preference for vegetables and fruits and difficulties in preparing and cooking those foods, and high preference for and habits of consuming sugar-sweetened foods and beverages. A similar division did not emerge on child-related barriers, and the items evaluating the child’s interest and request for foods with added sugars dropped during the factorial analysis. The literature indicates that parents experience this type of barrier even with young children; recent findings have shown that children’s demands and difficulty in establishing rules for sugary and caloric food intake are the main parental challenges when promoting a healthy diet. For instance, a qualitative study concluded that child behavioral factors were the most frequent barriers mentioned by parents to promoting healthy eating habits in their children up to the age of four, which included several references to the child’s preference for treats and snacks and eating self-regulation issues [37]. Similar findings were also found by Magalhães and colleagues in a Portuguese sample of 9 to 14-year-old children [38]. When asked about the main barriers to adopting and maintaining a healthy diet, most children identified themselves and their characteristics as an obstacle, particularly their preferences for and habits of ingesting high-calorie foods, difficulty in self-regulating to resist temptations, and the lack of motivation to eat healthy foods [38]. It is possible, though, that, for cultural reasons, this type of difficulty only manifests in Portuguese parents at a later stage of their children’s life. These findings might also be explained by some inconsistency of parents of young children on identifying sugar consumption as a problem or not considering reducing sugar as a relevant goal to pursue when improving a young child’s diet. As such, it is necessary to continue efforts to develop measures that can assess in detail which challenges are being faced by parents regarding children’s unhealthy food intake or difficulties in self-regulating their food intake during the first years of life.

The convergent/discriminant and known-group validity studies partially validated the hypothesis initially drawn. Only the subscales referring to child- and parent-related barriers (vegetables and fruits) were negatively associated with food parenting self-efficacy, with moderate and weak correlations, respectively. Our findings suggest that individual preferences regarding fruit and vegetables might impact more on the parental ability to promote overall good eating habits in their children, compared with contextual barriers. We also found weak and negative relations between child-related barriers and feeding practices to promote children’s eating self-regulation [25]. A possible explanation for our correlational findings is that parents might consider strategies focused on promoting the child’s self-regulation as not very effective or take longer to have the desired impact on children who are more selective and less interested in healthy foods. The previous literature showed that caregivers frequently pressure the child to eat more or insist that they eat a less preferred food to deal with children’s food avoidance behaviors in the first years of life [39,40]. Parents may also have more difficulties in relying on their child’s ability to accurately report their satiety and hunger sensations when children are younger and with eating behavior issues, which can discourage the use of this type of feeding practice [41,42].

Regarding parental barriers, our results showed that parents who do not like vegetables and fruit and have difficulty preparing these foods tend to use overall fewer strategies to promote their children’s eating self-regulation. We also found a weak and negative correlation between parental-related barriers related to added sugars and the use of teaching strategies about the consequences of eating specific foods. The use of such child-centered feeding strategies requires a greater effort from parents to recognize and respond to their child’s satiety and hunger cues, maintain a positive emotional climate during the meal, and consistently use responsive verbalizations and practices [43,44]. This attitude may be more difficult to adopt when parents have less healthy preferences, are less motivated to eat healthily, or are less used to self-regulating their own eating behavior. 

As hypothesized, parents who identified their children as having an easy or well-regulated temperament reported significantly fewer child-related barriers than those who perceived their children as poorly self-regulated/under-controlled or inhibited. Specific child temperament characteristics, such as negative affect or emotionality [27,45], surgency, and effortful control [45], have been linked to food avoidance behaviors. Children with an easier temperament may have higher levels of food self-regulation and be less triggered by specific feeding contexts [26], and therefore present less challenging and easier-to-manage eating behaviors.

This study has some limitations. The data were collected regionally, and the sample does not represent the Portuguese population. The social desirability effect must be considered since all instruments were completed by parents. The second study data collection was performed during the COVID-19 pandemic, which might have changed the feeding context of the families regarding children’s food requests, feeding practices used by parents to maintain healthy eating habits and other perceived barriers related to food access and cost [46,47]. Also, most mothers in both studies had a higher education level. Earlier studies [18] found that context-related barriers are more common in families with unfavorable socioeconomic conditions and low levels of education, leading families to choose less expensive and less healthy foods.

## 6. Conclusions

The present work about the development and psychometric features of the Parental Perception of Children’s Healthy Feeding Barriers Questionnaire confirmed the overall validity and reliability of the instrument, making it suitable for use in primary cross-sectional and interventional studies and as a first approach to the subject in clinical contexts. The questionnaire’s structure highlights the main categories found in primary studies about barriers experienced by parents when promoting healthy eating habits in their young children: individual (child and parents) and contextual barriers. However, we recognize its limitations regarding evaluating parental barriers related to children’s cravings and demands for sugar-sweetened foods, already evident in preschool age. This instrument would also benefit from further confirmatory factorial analysis and measurement invariance studies with a more diversified sample. The convergent and discriminant validity analysis showed interesting results that help clarify the relationships between different types of barriers perceived by parents and parental self-efficacy in promoting healthy eating habits, including the child’s temperament and parental practices to encourage children’s eating self-regulation practices.

## Figures and Tables

**Table 1:** Exploratory factorial analysis of the instrument (*N* = 278).

Items	Factor 1. Child-Related Barriers	Factor 2. Parent-Related Barriers—Vegetables and Fruit	Factor 3. Parent-Related Barriers—Added Sugars	Factor 4. Social Context-Related Barriers	Factor 5. Cost-Related Barriers
The child does not like to try new vegetables.	0.84				
Getting my child to eat vegetables at meals is difficult.	0.82				
My child doesn’t like the taste of vegetables.	0.81				
My child does not like the texture of vegetables.	0.80				
My child is a picky eater.	0.71				
The child does not like to try new fruits.	0.71				
My child does not like the texture of fruits.	0.49				
Getting my child to eat fruit with meals is difficult.	0.48				
The child does not like plain milk or plain yogurt.	0.39				
I don’t like fruits myself.		0.76			
I don’t know how to cook vegetables.		0.72			
I don’t like vegetables myself.		0.69			
It is difficult to find recipes for vegetables.		0.63			
Preparing the fruit to eat (peeling, cutting) is much work.		0.60			
Preparing vegetables the child likes takes much time.		0.56			
I like having cakes, sweets, or treats at home.			0.77		
I have a sweet tooth.			0.76		
I really like going to the cafe to eat cakes or sweets.			0.71		
It is very difficult to resist buying cakes, sweets, or treats because they are always visible in stores and shopping centers.			0.59		
I like to make the child happy by buying him a cake, candy, or a treat when we go out.			0.46		
At school, they offer cakes, sweets, or treats to children.				0.71	
The child’s friends eat unhealthy food around them.				0.70	
The food offered at school does not provide healthy food choices.				0.65	
When the child goes to birthday parties, they always bring home many goodies.				0.58	
The child sees other family members eating unhealthy foods.				0.51	
Grandparents, other family members, or friends often offer you cakes, sweets, or treats.				0.44	
Fruit is expensive					0.91
Vegetables are expensive					0.90
% Explained variance	16.33	11.76	9.08	8.30	7.44
Eigenvalue	5.86	2.84	2.42	1.99	1.71

**Table 2:** Correlations between the Parental Perception of Children’s Healthy Feeding Barriers Questionnaire dimensions: food parenting self-efficacy and parental practices to promote the child’s eating self-regulation (*N* = 168).

Variables	Child-Related Barriers	Parent-Related Barriers—Vegetables and Fruit	Parent-Related Barriers—Added Sugars	Social Context-Related Barriers	Cost-Related Barriers
Food parenting self-efficacy	−0.460 **^a^	−0.269 **^a^	−0.114^a^	−0.044 ^a^	−0.110 ^b^
Teaching about eating consequences	−0.190 *^b^	−0.188 *^b^	−0.202 **^b^	−0.011 ^b^	−0.028 ^b^
Prompting for eating self-regulation	−0.223 **^a^	−0.211 **^a^	−0.146^a^	−0.113 ^a^	−0.002 ^b^

Note. ^a^ Pearson correlation coefficient, ^b^ Spearman correlation coefficient. * *p* < 0.05, ** *p* < 0.01.

## Data Availability

The data and analysis performed in this study are available via an e-mail request to the authors; a formal data sharing agreement will be required.
